# Reviewed article: Silva TFPLA, Peixoto HM, Freitas LRS, Araújo ELL, Ramalho WM. Trends in dengue incidence and lethality: interrupted time series analysis, Brazil, 2001-2022. Epidemiol Serv Saude. 2025:34;e20240424.

**DOI:** 10.1590/S2237-96222025v34e20240424.b

**Published:** 2025-09-08

**Authors:** Leonardo Trench

**Affiliations:** 1 USP FOB, Saúde Coletiva

**Table:** 

Rating	Excellent	Good	Average	Below average	Poor
Interest		✓			
Quality			✓		
Originality			✓		
Overall			✓		

**Table:** 

Questionnaire	Yes	No	Not applicable
Does the manuscript contain new and significant information to justify publication?	✓		
Does the Abstract (Summary) clearly and accurately describe the content of the article?	✓		
Is the problem significant and concisely stated?		✓	
Are the methods described comprehensively?	✓		
Are the interpretations and conclusions justified by the results?	✓		
Is adequate reference made to other work in the field?	✓		

## Manuscript Structure

Length of article is: Adequate

Number of tables is: Adequate

Number of figures is: Adequate

Please state any conflict(s) of interest that you have in relation to the review of this paper (state “none” if this is not applicable): None

## Comments

É um trabalho bastante interessante para o SUS, visto que estamos falando de uma doença que não respeita fronteiras sócio econômicas nem culturais, com grandes prejuízos, de maneira geral para toda a sociedade e para o sistema como um todo.

A forma escrita como os ricos resultados são apresentados dificulta o entendimento do leitor, creio que se utilizassem gráficos ao invés de somente tabelas, e se o relato por escrito trouxesse apenas os resultados mais relevantes, seria mais interessante, tornando a leitura mais prazeirosa e eficaz.

Para um estudo com tantos resultados, pense na possibilidade de trabalhar melhor com gráficos, infográficos. Outro fato importante quanto às tabelas e figuras é que:

Tabelas devem ter suas legendas acima, mas figuras as legendas são abaixo delas, porém, o que é apresentado como figura 1 e 2, na verdade são tabelas e deveriam estar representados como tal.

Penso que a pergunta da pesquisa e as hipóteses poderiam estar mais bem detalhadas, facilitando as interpretações doa análises, que por não serem corriqueiras podem trazer dificuldades de entendimento aos menos familiarizados com elas.

Quanto aos métodos, creio ser interessante incluir a referência das notas ministeriais que justificam o uso de casos prováveis, ao invés de casos comprovados nos cálculos de incidência e letalidade, bem como sua explicação breve no texto.

As conclusões estão bem sustentadas pelos resultados, mas entendo que este estudo não encerra a discussão, devendo abrir a possibilidade de mais estudos, além do que, entendo que seriam importantíssimo relatar mais claramente a importância dele para o sistema e para a comunidade, como gestores e tomadores de decisões devem ser alertados sobre o assunto em questão.

Quanto às palavras chave, eu trocaria Mortalidade por registros de mortalidade

Destaca-se que a metodologia Análise de Séries Temporais Interrompida para o assunto, torna o trabalho inovador e motivador para outras análises do mesmo tipo, com dados interessantes que facilmente podem ser utilizados pela gestão de uma maneira geral se bem explicados, contempla, desta forma todos os requisitos necessários, porém a forma de descrever os resultados e discuti-los torna cansativo e improdutivo, como já relatei acima.

Algumas observações quanto a digitação estão colocadas abaixo

Pagina 1 linha 41: substituir o verbo “é” para o plural “são”.

Página 2 linha 46: Excluídas

Pagina 2 linha 45: usar ponto ou deixar este dado para os critérios de exclusão

Linha 10 pagina 4: Especificar os CIDS relacionados aos óbitos em questão

Tabelas devem ter a legenda acima e figura a legenda abaixo

Por que a figura 1 e 2 são figuras e não tabelas?

Linha 38 página 4. Não passavam de 600/100.000 habitantes por macrorregião, discordo quanto a ser sudeste e centro oeste as principais macrorregiões, pois isso ocorre apenas nos 3 primeiros anos e se somar os cinco primeiros, centro oeste e norte passam sudeste, portanto, este paragrafo merece ser melhor escrito

A partir da linha 40 os resultados expostos não condizem com a tabela chamada de figura: por exemplo em 2001 e 2023 não são essas as macrorregiões que apresentam menores e maiores índices. Sugiro reescrever o paragrafo.

Inclusive, como são trazidos muitos resultados, sugiro apresentar por escrito os mais relevantes e fazer um gráfico de colunasou linhas específicas para cada macrorregião para apresentação do geral, desta maneira está muito difícil de acompanhar a relevância dos resultados que são importantes.

A tabela um não traz os dados consolidados como são apresentados no texto, seria melhor se aparecesse desta maneira

